# Supersaturated Oxygen Therapy as a Treatment for No Reflow

**DOI:** 10.1016/j.jaccas.2024.103102

**Published:** 2025-02-05

**Authors:** Megha Prasad, Natali Sorajja, Sandeep Nathan, Jeffrey Chambers

**Affiliations:** a Department of Medicine, Columbia University Irving Medical Center/NewYork-Presbyterian Hospital, New York, New York, USA; b Albert Einstein College of Medicine, Bronx, New York, USA; c University of Chicago Pritzker School of Medicine, Chicago, Illinois, USA; d Metropolitan Heart and Vascular Institute, Minneapolis, Minnesota, USA

**Keywords:** complication, myocardial ischemia, percutaneous coronary intervention, treatment

## Abstract

No reflow, an interruption in epicardial and microvascular blood flow, during percutaneous coronary intervention (PCI) is associated with adverse outcomes but continues to have limited therapeutic options. We present a case of a patient with multiple comorbidities, multivessel disease and reduced left ventricular function with calcified left anterior descending stenosis who was treated with rotational atherectomy, complicated by slow flow after balloon dilatation. Infusion of supersaturated oxygen (SSO_2_) into the left main was instituted as an adjunct to PCI, along with pharmacologic vasodilators. Subsequently, there was resolution of the patient’s symptoms and improvement in ejection fraction postprocedurally. The potential role of SSO_2_ in treating patients with intraoperative no reflow is intriguing, given no reflow’s current limited treatment options and known increased risk of adverse events (major adverse cardiovascular events, cardiogenic shock, and so on). SSO_2_ may be a promising therapy for PCI complicated by no reflow.

## Visual Summary

**Figure:**
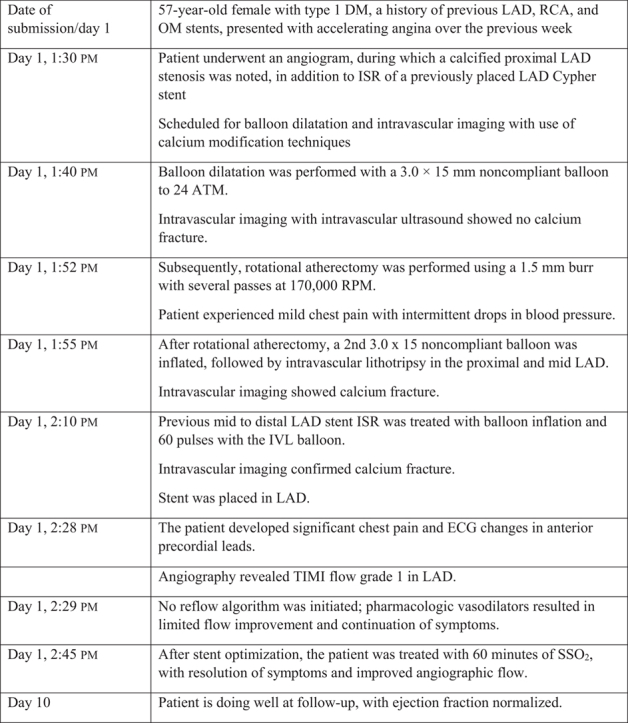

**Visual Summary:** Timeline of Events

Day of Submission	57-Year-Old Female With Type 1 DM, A History of Previous LAD, RCA, and OM Stents, Presented With Accelerating Angina Over the Previous Week
Day 1, 1:30 pm	Patient underwent an angiogram, during which a calcified proximal LAD stenosis was noted, in addition to ISR of a previously placed LAD Cypher stent Scheduled for balloon dilatation and intravascular imaging with use of calcium modification techniques
Day 1, 1:40 pm	Balloon dilatation was performed with a 3.0 × 15 mm noncompliant balloon to 24 ATM. Intravascular imaging with intravascular ultrasound showed no calcium fracture.
Day 1, 1:52 pm	Subsequently, rotational atherectomy was performed using a 1.5 mm burr with several passes at 170,000 RPM. Patient experienced mild chest pain with intermittent drops in blood pressure.
Day 1, 1:55 pm	After rotational atherectomy, a 2nd 3.0 x 15 noncompliant balloon was inflated, followed by intravascular lithotripsy in the proximal and mid LAD. Intravascular imaging showed calcium fracture.
Day 1, 2:10 pm	Previous mid to distal LAD stent ISR was treated with balloon inflation and 60 pulses with the IVL balloon. Intravascular imaging confirmed calcium fracture. Stent was placed in LAD.
Day 1, 2:28 pm	The patient developed significant chest pain and ECG changes in anterior precordial leads. Angiography revealed TIMI flow grade 1 in LAD.
Day 1, 2:29 pm	No reflow algorithm was initiated; pharmacologic vasodilators resulted in limited flow improvement and continuation of symptoms.
Day 1, 2:45 pm	After stent optimization, the patient was treated with 60 minutes of SSO_2_, with resolution of symptoms and improved angiographic flow.
Day 10	Patient is doing well at follow-up, with ejection fraction normalized.

## Case Presentation

**Figure 1:**
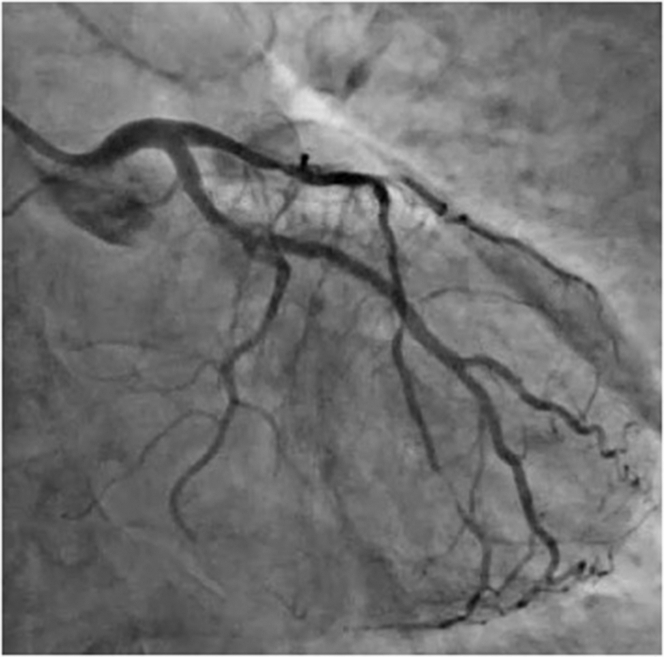
Angiogram Demonstrating Calcified Proximal Left Anterior Descending Stenosis and In-Stent Restenosis of Previously Placed Stent

We present a case of a 57-year-old woman with type 1 diabetes mellitus, and a history of previous left anterior descending (LAD), right coronary artery (RCA), and obtuse marginal stents who presented with accelerating angina over the last week. Echocardiogram showed an ejection fraction of 45% with mild apical hypokinesis. The patient was scheduled for an angiogram during which a calcified proximal LAD stenosis was noted in addition to in-stent restenosis (ISR) of previously placed mid LAD Cypher stent (Figure 1). The procedural plan was balloon dilatation and intravascular imaging with use of calcium modification techniques as indicated.

**Take-Home Messages**

• This case highlights the usage of SSO_2_ as a treatment for symptom solution and ejection fraction normalization in a PCI case complicated by slow flow
• There are currently limited treatment options for no reflow in the cardiac catheterization laboratory, thus marking SSO_2_ as a promising and intriguing therapy

## Management

**Figure 2:**
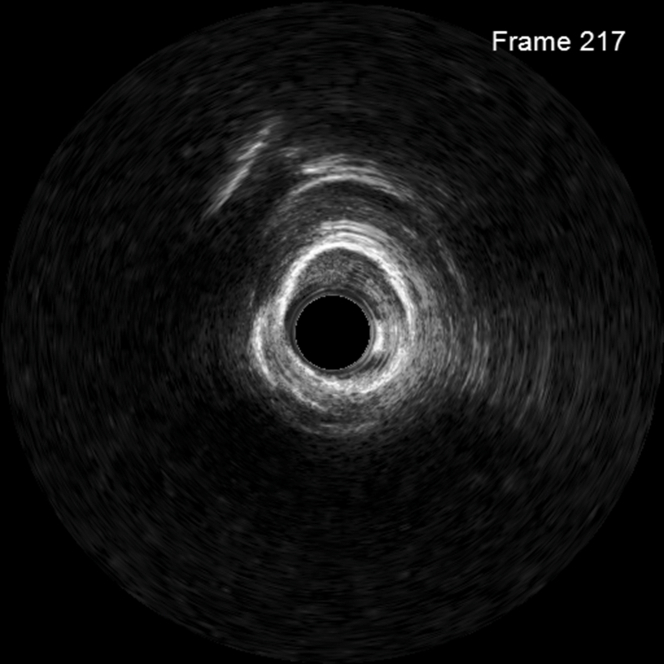
Intravascular Ultrasound Demonstrating No Calcium Fracture

**Figure 3:**
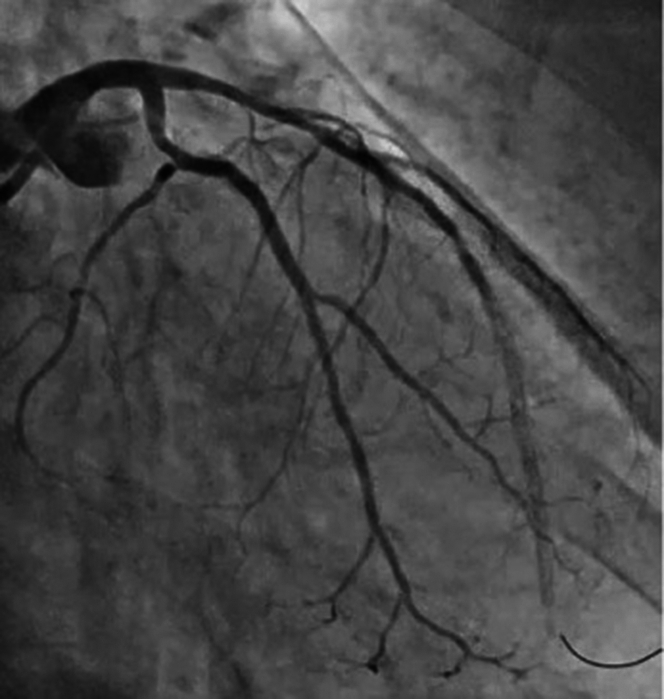
Angiogram Demonstrating TIMI Flow Grade 1 in Left Anterior Descending Artery

**Figure 4:**
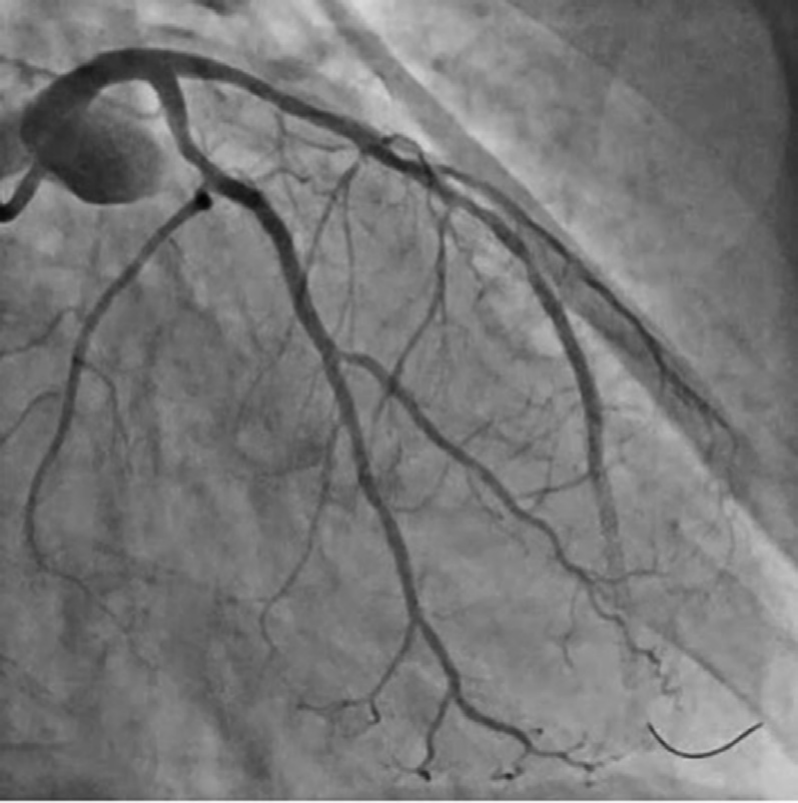
Angiogram After No-Reflow Algorithm and Vasodilators

**Figure 5:**
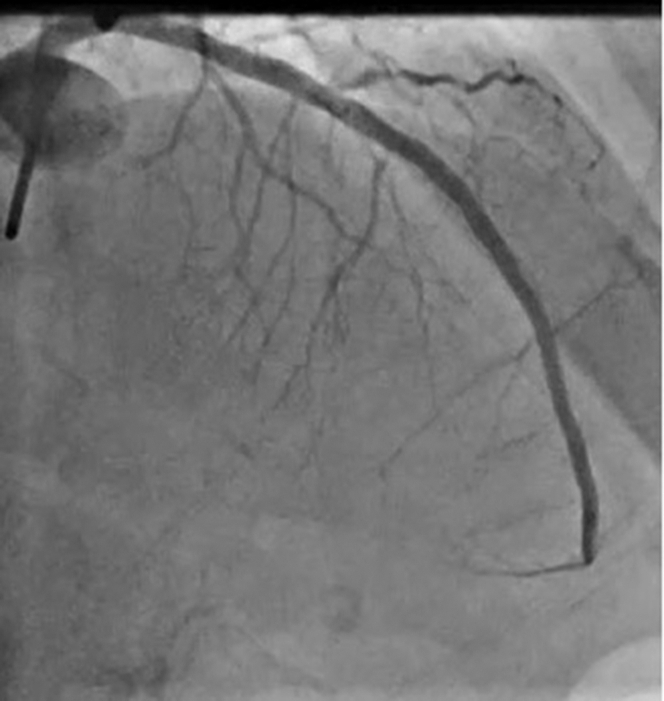
Angiogram After SuperSaturated Oxygen Therapy

Initial balloon dilatation was performed with a 3.0 × 15 mm noncompliant balloon to 24 atm. Intravascular imaging with intravascular ultrasound was performed, which showed no calcium fracture (Figure 2), prompting plan for rotational atherectomy. A 1.5-mm burr was used with several passes at 170,000 rpm. The patient had intermittent drops in blood pressure with mild chest pain. After rotational atherectomy, a second 3.0 × 15 mm noncompliant balloon was inflated, followed by intravascular lithotripsy in the proximal and mid LAD, and intravascular imaging was performed which showed calcium fracture. Previous mid to distal LAD stent ISR was treated with balloon inflation and 60 pulses with the intravascular lithotripsy balloon. Stent was placed in LAD after confirming calcium fracture on intravascular ultrasound. The patient then developed significant chest pain with ECG changes in the anterior precordial leads. Angiography revealed TIMI flow grade 1 in the LAD (Figure 3). No reflow algorithm was followed with pharmacologic vasodilators including nipride with limited improvement in flow but continued symptoms (Figure 4). After stent optimization, the patient was treated with 60 minutes of SSO_2_ therapy with resolution in symptoms and improvement in angiographic flow (Figure 5). At follow-up 10 days postsurgery, the patient is doing well, with ejection fraction having returned to normal. Detailed information about equipment used in this procedure is located in Table 1.

**Table 1:** Equipment List

• 3.0 x 15 mm noncompliant balloon (2)
• Intravascular lithotripsy balloon
• 1.5 mm burr
• Stent (placed into LAD)
• TherOx Super Saturated Oxygen (SSO_2_) therapy
• 6-F EBU3.5 guide catheter
• Micropuncture kit
• Sion blue wire

## Discussion

**Figure 6:**
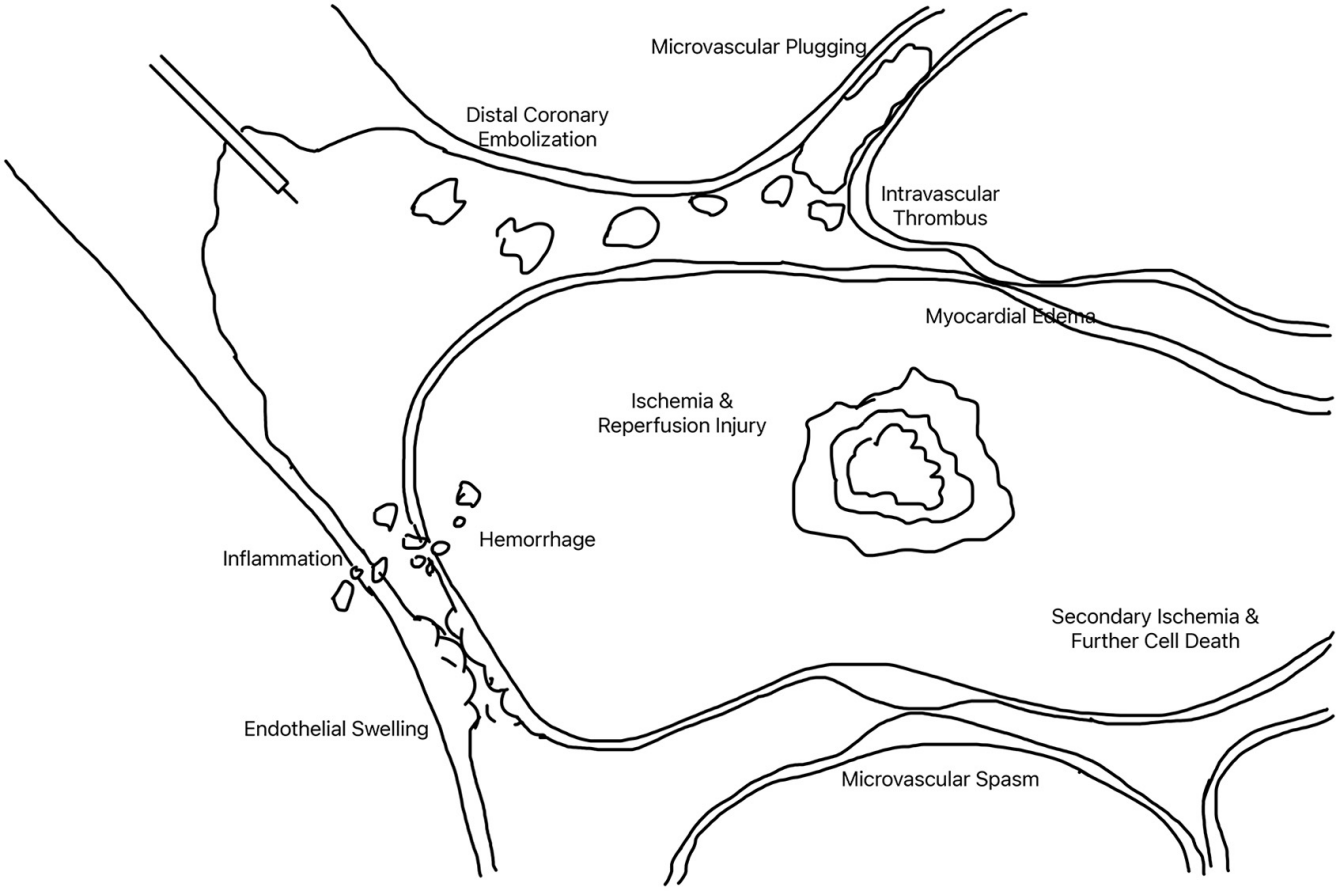
4 Major Pathophysiologic Mechanisms Underlying No-Reflow

No reflow may occur during PCI and is characterized by angiographic evidence of low flow or cessation of epicardial flow in the absence of mechanical obstruction resulting in myocardial ischemia. Structural and functional coronary microcirculation alteration is a widely recognized underlying cause. As we prevent and treat no-reflow phenomenon, and attempt to reduce associated morbidity and mortality, it is important to understand the underlying pathophysiology which begins at the level of the microcirculation.^1^^,^^2^ Four underlying mechanisms have often been cited that may result in no reflow: distal atherothrombotic embolization, ischemic damage, reperfusion injury, and individual susceptibility to microvascular damage (Figure 6).^3^^,^^4^ Structural damage to the microvasculature resulting from coronary occlusion with cessation and then restoration of epicardial blood flow may prevent adequate blood flow at the myocyte level, leading to scar.^3^ Although we may restore epicardial patency, data suggest that no reflow is a more complicated process driven by microvascular abnormalities. Cardiac myocyte microscopic examination from areas of no reflow were characterized by capillary endothelial damage, significant edema, and intravascular plugging by leukocytes, platelets, and fibrin, not dissimilar from patients with acute coronary syndrome.^3^ Similar to a myocardial infarction, no reflow may result in significant symptoms of ischemia, as well as reduction in ejection fraction, negative remodeling, and adverse outcomes. Although there are a variety of no reflow algorithms focusing on pharmacological therapies to treat no reflow, these medications focus on vasodilatation of the microcirculation. Certain patient substrates and procedures may be more prone to no reflow during a PCI, including patients with baseline microvascular dysfunction as well as those patients with extensive calcium and/or thrombus who may have an increased risk of intraprocedural distal embolization.^1^^,^^4^

Despite the complex underlying pathophysiology of no reflow, our current approach to management largely centers around restoration of epicardial flow with little attention given to the microcirculation in part caused by limited therapies that may treat this condition.^5^ Because microvascular damage occurs in a zone of myocardial necrosis, treating no reflow with a focus on epicardial patency may have a limited role in reducing infarct size, and subsequent infarct expansion and remodeling may occur. A shift to focus on adjunctive management of microcirculatory changes may provide improved options for patients with no reflow in the cardiac catheterization laboratory.

SSO_2_ is a U.S. Food and Drug Administration–approved adjunctive therapy for patients with anterior wall myocardial infarction that has been shown to be associated with significant reduction in infarct size, improved regional myocardial blood flow, and improvement in ejection fraction potentially caused by less adverse remodeling. Mechanistically, this is thought to be caused by relief of microvascular obstruction and improvement in microvascular dysfunction that may not be treated with simple reperfusion strategies focusing on epicardial patency. Pathophysiologically, an anterior ST-segment elevation myocardial infarction and no reflow in the LAD especially may have similar myocyte and myocardial repercussions, and such an adjunctive treatment of microcirculatory plugging and dysfunction may be beneficial in patients with no reflow. Given the similar underlying downstream myocardial damage caused by an anterior myocardial infarction and no reflow, SSO_2_ may have a role as an adjunctive technology in these clinical scenarios. Especially as the age of the average patient presenting to catheterization laboratory increases, with an increasing prevalence of complex lesion subsets and higher-risk patients who may be more prone to no reflow, it is critical to evaluate technologies that may provide additional benefit to reduce adverse outcomes in these patients. Our case illustrates the safe and potentially useful role of SSO_2_ in patients with no reflow.

## Conclusions

To our knowledge, this is the first case of SSO_2_ being used to treat a patient with no reflow. This case describes successful treatment with SSO_2_ therapy and resolution of flow of LAD in a patient with calcified disease, and no reflow after atherectomy. Future studies are needed to better understand the role of SSO_2_ as an adjunctive therapy in patients with no reflow.

## Funding Support and Author Disclosures

This research did not receive any specific grant from funding agencies in the public, commercial, or not-for-profit sectors. Dr Prasad has served on the Speakers Bureau/as a consultant to Boston Scientific, Boehringer Ingelheim, Abbott, CONAVI, Chiesi, Shockwave Medical, and Abiomed. Dr Chambers has served as a consultant to Zoll. Dr Nathan has served as a consultant to Zoll; and is co-national principal investigator for the SSCORE Registry Ms Sorajja has reported that she has no relationships relevant to the contents of this paper to disclose.
